# Relationship between clinical and instrumental balance assessments in chronic post-stroke hemiparesis subjects

**DOI:** 10.1186/1743-0003-10-95

**Published:** 2013-08-13

**Authors:** Zimi Sawacha, Elena Carraro, Paola Contessa, Annamaria Guiotto, Stefano Masiero, Claudio Cobelli

**Affiliations:** 1 Department of Information Engineering, University of Padova, Padova, Italy; 2 Department of Rehabilitation Medicine, University of Padova, Padova, Italy

## Abstract

**Background:**

Stroke is often associated with balance deficits that increase the risk of falls and may lead to severe mobility disfunctions or death. The purpose of this study is to establish the relation between the outcome of instrumented posturography and of the most commonly used clinical balance tests, and investigate their role for obtaining reliable feedback on stroke patients’ balance impairment.

**Methods:**

Romberg test was performed on 20 subjects, 10 hemiplegic post-stroke subjects (SS, 69.4 ± 8.2 years old) and 10 control subjects (CS, 61.6 ± 8.6 years old), with 1 Bertec force plate. The following parameters were estimated from the centre of pressure (CoP) trajectory, which can be used to define subjects’ performance during the balance task: sway area; ellipse (containing 95% of the data); mean CoP path and velocity in the anterior-posterior and medio-lateral directions. The following clinical scales and tests were administered to the subjects: Tinetti Balance test (TB); Berg Balance test (BBT); Time up and go test (TUG), Fugl-Meyer (lower limbs) (FM), Motricity Index (lower limbs), Trunk Control Test, Functional Independence Measure. Comparison between SS and CS subjects was performed by using the Student t-test. The Pearson Correlation coefficient was computed between instrumental and clinical parameters.

**Results:**

Mean ± standard deviation for the balance scales scores of SS were: 12.5 ± 3.6 for TB, 42.9 ± 13.1 for BBT, 24 s and 75 cent ± 25 s and 70 cent for TUG. Correlation was found among some CoP parameters and both BBT and TUG in the eyes open and closed conditions (0.9 ≤ R ≤ 0.8). Sway area correlated only with TUG. Statistically significant differences were found between SS and CS in all CoP parameters in eyes open condition (p < 0.04); whereas in eyes closed condition only CoP path and velocity (p < 0.02) differed significantly**.**

**Conclusions:**

Correlation was found only among some of the clinical and instrumental balance outcomes, indicating that they might measure different aspects of balance control. Consistently with previous findings in healthy and pathological subjects, our results suggest that instrumented posturography should be recommended for use in clinical practice in addition to clinical functional tests.

## Background

Stroke is the third leading cause of death and the major cause of severe disability and impairment in the industrialized world [1]. In Europe, about 250 strokes/100.000 inhabitants occur every year, with a rising trend [2]. Following a stroke, patients frequently suffer severe disability and marked limitations in activities of daily living. Postural instability is one of the major deficits following a stroke, with associated increased risk of fall; a consequence of this problem is reduced mobility, increased disability and even mortality [3-8]. Stroke subjects who retain the ability to stand show delayed and disrupted equilibrium reactions, exaggerated postural sway in both sagittal and frontal planes, reduced weight-bearing on the paretic limb and increased risk of falling [9]. The clinical and social impact of postural instability has produced a great deal of research in this field that allowed the development of several functional tests and laboratory methods (posturography) to explore the extent of balance dysfunction [10].

Both these functional test and posturographic techniques have been applied to specifically investigate balance deficits in stroke patients [11,12]. Quantitative posturography utilizes force plates to monitor the trajectory of the centre of pressure (CoP). The CoP trajectory reflects the body sway during standing and the ability of the nervous and musculoskeletal systems to integrate information from multiple sensory systems, including the visual, the somatosensory, and the vestibular system to maintain balance [13,14]. Alterations of the postural control system are reflected in changes of CoP characteristics and parameters [13,14], which is therefore a key variable for monitoring the postural control system [13-16]. Although instrumented posturography has demonstrated its validity in monitoring balance, the use of force plates in the clinical practice is not yet common and simple test batteries and questionnaires to test balance and mobility are often employed as useful alternatives [7,11,17]. Some of these tests and scales, which are described in detail in the Methods section, include the Fugl-Meyer scale (FM) [18]; the lower Motricity Index (lo-MI) [19]; the Trunk Control Test (TCT) [20]; the Functional Independence Measure (FIM) [21-24]; the Tinetti Balance scale (TB) [7]; the Berg Balance Test (BBT) [24]; and the Time up and go Test (TUG) [17]. Some of these tests have proved to be a valid and reliable indicator for balance ability [12]. For instance, in a study by Bogle et al. (1996), falls in stroke patients were associated with poor performance in the Berg Balance Scale [25]. However, the individual clinical functional tests do not reflect the complexity and multidimensional nature of balance [26].

While both functional tests and instrumented measures are used to monitor balance function in stroke subjects, with the clinical settings relying mostly on the former, their relationship and their usefulness as means for obtaining reliable feedback on the patient balance impairments and for evaluating the effects of a rehabilitative treatment has not been investigated yet. Therefore, the aim of this study is to assess postural stability using both computerised posturography and functional balance tests in chronic post-stroke patients and to investigate their relation.

## Methods

### Participants

20 subjects participated in the study, 10 control subjects (CS) and 10 hemiplegic post-stroke subjects (SS).

SS patients were recruited from the outpatient clinic of the Rehabilitation Department of the University of Padova (Italy). All patients were diagnosed with chronic post-stroke hemiplegia/hemiparesis (> 1 year from onset) and were able to walk independently or with supervision (Functional Ambulation Classification scores ≥3) [27]. Exclusion criteria for SS were: concomitant cardiovascular disease; other neurological or psychiatric diseases; severe visual or auditory impairments (reduced visual acuity was accepted if adequately corrected). Patients with multiple cerebrovascular lesions or with infratentorial lesion were not recruited. Patients were also excluded if their pharmacological therapy changed during the trial or in the previous month; or if they attended a rehabilitation treatment during the study or in the 3 months before the study.

The CS consisted of healthy subjects enrolled among hospital personnel. The study was approved by the ethics committee of the Hospital of Padova (Italy). An informed consent form was obtained from all participants.

SS and CS subject groups were matched for age and BMI. Mean age was 69.4 ± 8.21 years for SS and 61.60 ± 8.57 years for CS (p = 0.058); mean BMI was 25.16 ± 2.48 kg/m^2^ for SS and 27.30 ± 2.24 kg/m^2^ for CS (p = 0.066). Body mass and height did not differ: mean body mass for SS and CS was 80.00 ± 12.26 kg and 80.30 ± 8.12 kg, respectively (p = 0.950); mean height was 177.56 ± 9.08 cm and 172.30 ± 5.43 cm, respectively (p = 0.140). The time since stroke for the SS group was on average 7.5 ± 8.9 years.

### Clinical and instrumental evaluation

Instrumental evaluation consisted in the Romberg test, which was performed on all subjects with 1 Bertec force plate (FP4060-10, 960 Hz). Subjects were asked to stand on the force plate, with their feet placed so as to maintain the heels together and a 30 degrees angle between the right and left toes, and to relax the arms along the body [28]. To ensure similar angles between the feet throughout the test, a guide made of heavy cardboard was placed on the force plate, and the subjects lined their feet up along both arms of the foot-guide. Once the subjects assumed the correct posture, they were asked to maintain the upright standing position for 60 s with their eyes open (EO) while looking at a circular target placed at a distance of 4 m in front of them and then to maintain the same position for 60 s with their eyes closed (EC) [29]. The CoP trajectory was acquired during the Romberg test. The signal underwent a post-acquisition filtering and downsampling technique, thus reducing the frequency to 100 samples/s (the first 20 s of the signal were not analyzed) [13,14,18,29] From the CoP signals the following posturographic parameters were computed [13,14,18,29]: the sway area, which is a measure of the area included in CoP displacement per unit of time (mm^2^/s); the ellipse containing 95% of the CoP data point; the CoP path, calculated as the total length of the CoP path; the CoP path in both in the anterior-posterior (AP) and in the medio-lateral (ML) directions, which are approximated by the sum of the distances between consecutive points in the AP and ML directions; and the CoP velocity (CoPv), as well the CoP velocity in both the AP and in the ML directions. All data analysis was performed using the Matlab software.

The following clinical tests were administered exclusively to the SS subjects to quantify their motor and functional impairment and their degree of disability: Fugl-Meyer scale for lower limbs (FM); Motricity Index for lower limbs (lo-MI); Trunk Control Test (TCT); and Functional Independence Measure (FIM).

The FM scale is a multi-item Likert-type scale developed as an evaluative measure of recovery from hemiplegic stroke. We used the subscale for motor domain of the lower limbs that includes items quantifying movement, coordination, and reflex action about the hip, knee, and ankle; with motor score ranging from 0 (hemiplegia) to a maximum of 34 points (normal motor performance) [19]. lo-MI is an ordinal weighted scale used to assess the severity of motor impairment of the lower limb after a stroke. Essentially, it tests 6 limb movements while the patient is sitting on a chair or on the edge of the bed [20]. The Trunk Control Test evaluates three movements and one posture (balance in sitting position). The total score ranges from 0 to 100 points, a higher score indicating a better trunk performance [21]. FIM is a scale that measures the severity of disability and the outcomes of adult inpatient medical rehabilitation. It describes the level of independence on 18 items covering the domains of self-care, sphincter management, transfers, locomotion, communication, and social cognition. Each item is rated 1–7, with the higher rating indicating more independent performance. Total scores range from 18 to 126. The 13-item motor domain (range, 13–91) and the five-item cognitive domain (range, 5–35) are commonly scored separately [22-24,30].

The following clinical balance scales were administered to SS subjects to specifically evaluate their balance impairment: Tinetti Balance assessment tool (TB); Berg Balance Test (BBT); Time up and go Test (TUG).

The TB assessment tool is a simple, easily administered test that measures a patient’s gait and balance. Scoring is performed on a three point ordinal scale, ranging from 0 to 2. The individual scores are then combined to form three measures: an overall gait assessment score, an overall balance assessment score, and a gait and balance score [7]. In our work, we only considered the balance assessment score, with a maximum of 16 points. The BBT was developed to measure balance impairment among elderly people by assessing the performance of specific functional tasks. It’s a 14-item scale with a five-point scale, ranging from 0 to 4: ′0′ indicates the lowest level of function abilities and ′4′ the highest level of function abilities, with a total score of 56 [30]. The TUG test is a simple and quick functional mobility test that measures the time taken by an individual resting on a chair to stand in an upright position and to sit down again [17].

### Statistical analysis

Comparison between SS and CS subjects was performed by means of the Student t-test or Mann–Whitney U-test (SPSS v 13 Software), when appropriate based on the Levene’s Test for Equality of means. The Pearson Correlation coefficient was computed between instrumental and clinical balance parameters (SPSS v 13 Software). The threshold for statistical significance was set to p < 0.05.

## Results

Clinical measures are reported in Table 1 for SS subjects. Note that one of the subjects abandoned the study because his pharmacological therapy changed during the trial. Mean values for the balance scale scores were 12.5 ± 3.6 for TB, 42.9 ± 13.1 for BBT, 24 s and 75 cent ± 25 s and 70 cent for TUG, respectively.

**Table 1:** Clinical measurements for post-stroke subjects

**Subject #**	**1**	**2**	**3**	**4**	**5**	**6**	**7**	**8**	**9**	**10**
**MOTOR IMPAIRMENT**										
**Fugl-Meyer (leg)**	28	25	31	24	28	24	27	26	22	20
**Motricity Index (leg)**	99	64	99	83	36	83	91	91	59	72
**Trunk Control Test**	41	37	100	74	99	61	100	87	87	61
**Functional Indipendence Measure**	125	50	126	122	126	115	123	117	83	101
**BALANCE SCORES**										
**Time up and go (minutes/seconds)**	16′05	1′34′37	8′33	25′02	10′94	29′	9′09	10′36	29′09	8′45
**Berg Balance Test**	48	14	56	46	56	36	52	48	30	43
**Tinetti Balance**	14/16	6/16	16/16	11/16	16/16	12/16	16/16	16/16	9/16	9/16

Values for the clinical tests performed for post-stroke subjects. Note that only 9 subjects are reported because the pharmacological therapy of one subject changed during the trial.

**Figure 1:**
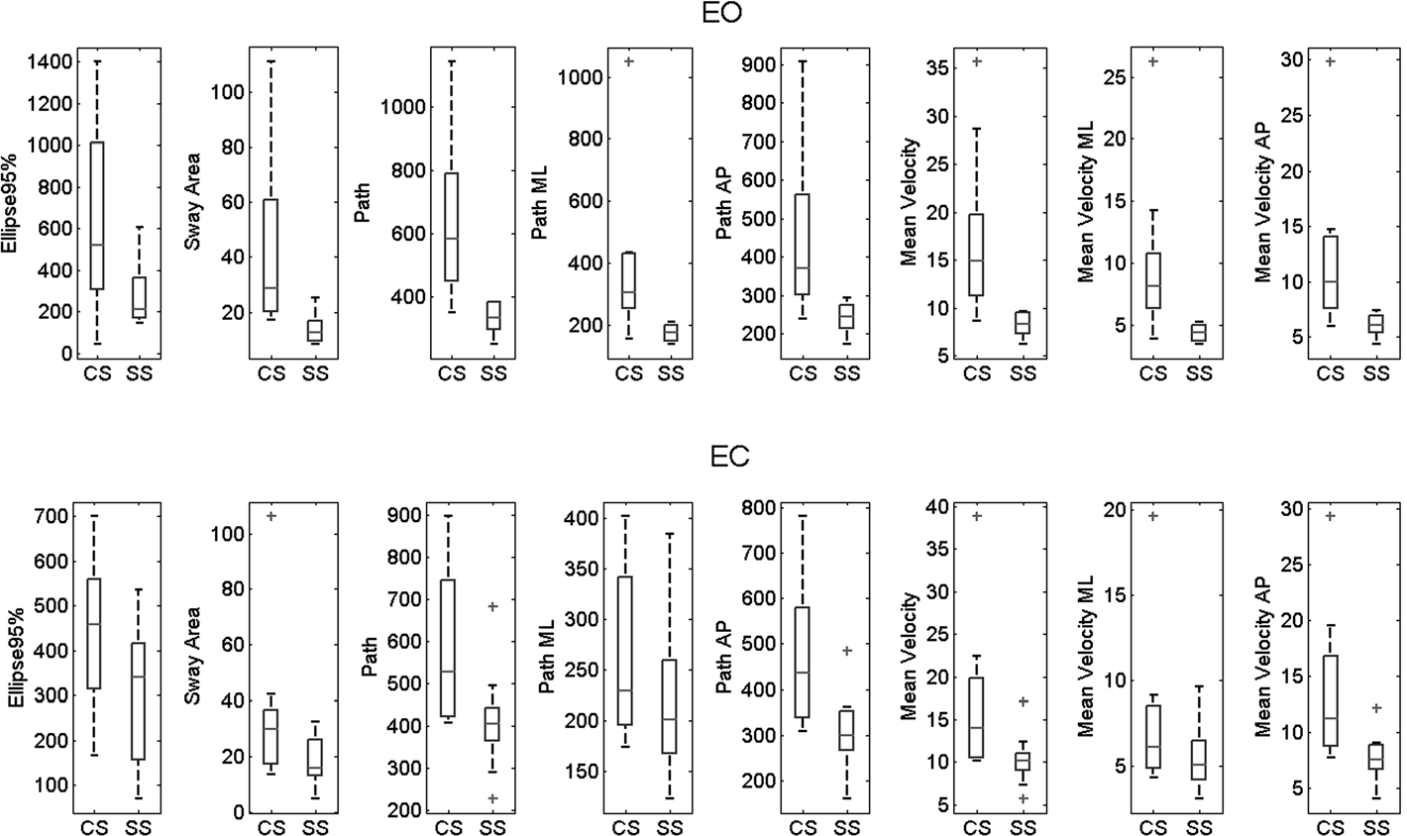
**Boxplots of the posturographic parameters.** Stroke subjects (SS) always on the right, Control Subjects (CS) always on the left. From left to right vertical axes represent: the ellipse 95% (Ellipse 95%), the sway area (Sway Area), the total path (Path), the path in medio-lateral direction (Path ML), the path in anterior-posterior direction (Path AP), the total mean velocity (Mean Velocity), the mean velocity in in medio-lateral direction (Mean Velocity ML), and the mean velocity in anterior-posterior direction (Mean Velocity AP). Both eyes open (EO) and eyes closed (EC) condition have been reported.

The CoP trajectory was computed from the force data acquired during the Romberg test in the EO and EC conditions. Results of all posturographic parameters are reported in Table 2 (EO) and Table 3 (EC) and in Figure 1 for SS and CS subjects, together with the p-value for significance. Statistically significant differences were found between SS and CS in all CoP parameters in EO condition (p < 0.05). In EC condition, significant differences were observed in the CoP path and in the CoP velocity and both the CoP path and velocity in the AP direction.

**Table 2:** Centre of pressure (CoP) parameters: Romberg test in eyes open (EO) condition

**CoP Parameters**	**SS**	**CS**	**P-values**
**Ellipse (mm**^**2**^**)**	647.9 ± 449.6	312.26 ± 168.5	0.042*
**Sway Area (mm**^**2**^**/s)**	43.6 ± 31.2	15.4 ± 7.4	0.005*
**Path (mm)**	665.1 ± 295.9	352.9 ± 82.6	0.005*
**Path ML (mm)**	384.9 ± 266.0	186.4 ± 43.4	0.032*
**Path AP (mm)**	444.4 ± 214	259.1 ± 62.9	0.018*
**CoPv (mm/s)**	17.80 ± 9.1	8.82 ± 2.1	0.007*
**CoPv ML (mm/s)**	10.1 ± 6.8	4.7 ± 1.1	0.022*
**CoPv AP (mm/s)**	12.1 ± 7.3	6.5 ± 1.6	0.031*

Parameters computed from the CoP trajectory acquired during the Romberg Test in the EO condition for post-stroke (SS, second column) and control subjects (CS, third column). All parameters were significantly different between SS and CS, as indicated by the p-values on the right-hand side column (the asterisks mark the values exceeding the threshold for significance, p < 0.05). Values are reported as mean ± standard deviation.

**Table 3:** Centre of pressure (CoP) parameters: Romberg test in eyes closed (EC) condition

**CoP Parameters**	**Stroke subjects**	**Control subjects**	**P-values**
**Ellipse (mm**^**2**^**)**	425.5 ± 180.5	309.3 ± 169.2	0.195
**Sway Area (mm**^**2**^**/s)**	38.3 ± 31.6	17.9 ± 8.4	0.067
**Path (mm)**	610.8 ± 192.2	412.7 ± 122.6	0.020*
**Path ML (mm)**	275.5 ± 86.1	215.80 ± 76.4	0.153
**Path AP (mm)**	494.4 ± 170.4	305.1 ± 85.5	0.008*
**CoPv (mm/s)**	18.2 ± 10.0	10.3 ± 3.1	0.033*
**CoPv ML (mm/s)**	8.4 ± 5.2	5.4 ± 1.9	0.117
**CoPv AP (mm/s)**	14.6 ± 7.6	7.6 ± 2.1	0.015*

Parameters computed from the CoP trajectory acquired during the Romberg Test in the EC condition for post-stroke (SS, second column) and control subjects (CS, third column). CoP path and velocity (CoPv), and the component in the antero-posterior direction were significantly different between SS and CS, as indicated by the p-values on the right-hand side column (the asterisks mark the values exceeding the threshold for significance, p < 0.05). Values are reported as mean ± standard deviation.

Regarding the correlation analysis results, it should be noticed that TB scores did not correlate with any instrumental measurements. Similarly, no correlation was observed among clinical scales and ellipse values, CoP path values, and CoP path values in the ML direction. In contrast, BBT was correlated with CoP path and CoPv in the AP direction in EO condition; with all CoPv-based parameters in EC condition. Similarly, moderate to high correlation was found, both in the EO and EC conditions, among TUG scores and sway area, CoP path in the AP direction and all CoPv-based parameters; with the only exceptions of CoP path in the AP direction with EC and CoPv in the ML direction with EO. See Table 4 for details.

**Table 4:** Correlations between clinical balance scales and laboratory measures

		**EO**			**EC**		
		**TB**	**BBT**	**TUG**	**TB**	**BBT**	**TUG**
**Ellipse (cm**^**2**^**)**	**R**^**2**^	0.08	-0.20	0.18	0.25	-0.24	0.47
	**p-value**	0.580	0.400	0.320	0.558	0.560	0.240
**Sway Area (cm**^**2**^**)**	**R**^**2**^	-0.15	-0.59	0.76	-0.25	-0.69	0.89
	**p-value**	0.470	0.090	0.011*	0.545	0.06	0.004*
**Path (cm)**	**R**^**2**^	-0.15	-0.25	0.33	-0.37	-0.49	0.50
	**p-value**	0.470	0.330	0.110	0.361	0.280	0.210
**Path ML (cm)**	**R**^**2**^	-0.17	0.15	-0.02	-0.42	-0.64	0.69
	**p-value**	0.450	0.380	0.670	0.299	0.080	0.060
**Path AP (cm)**	**R**^**2**^	-0.06	-0.74	0.86	-0.33	-0.33	0.37
	**p-value**	0.600	0.010*	0.001*	0.417	0.430	0.360
**CoPv (cm/s)**	**R **^**2**^	-0.18	-0.43	0.67	-0.32	-0.75	0.89
	**p-value**	0.430	0.146	0.034*	0.445	0.030*	0.003*
**CoPv ML (cm/s)**	**R**^**2**^	-0.19	0.09	0.09	-0.31	-0.79	0.93
	**p-value**	0.410	0.550	0.500	0.454	0.018*	0.001*
**CoPv AP (cm/s)**	**R **^**2**^	-0.10	-0.78	0.92	-0.32	-0.71	0.86
	**p-value**	0.540	0.010*	< 0.001*	0.430	0.040*	0.010*

R^2^-values and p-values for the correlation analysis between clinical measures (Tinetti Balance Test, TB; Berg-Balance Test, BBT; Time up and go Test, TUG) and laboratory measures (centre of pressure parameters, CoP) in the eyes open (EO) and eyes closed (EC) conditions for post-stroke subjects. Significant correlation is indicated with an asterisk (p < 0.05).

## Discussion

The main purpose of the study was to investigate the relation between the outcomes of instrumented posturography (the CoP parameters) and those of functional balance tests and scales in stroke subjects. Analysis of CoP components has proved to be useful in predicting the risk of falling and changes in postural performance [12-14,23,24,30-33] in healthy and pathologic subjects, and can detect changes in balance control produced by different treatments [12-16,31,32].

In terms of the outcomes of the posturographic analysis, our results showed statistical difference for all the parameters between healthy and stroke subjects in the EO condition, whereas only four parameters were statistically different between the two subject populations in the EC condition (CoP path and CoPv, AP CoP path and AP CoPv). The lack of significant difference in the EC condition should not be necessarily attributed to a worst performance of SS subjects in EO condition, but could simply reflect the decrease in balance control in EC condition for CS subjects, decrease that has been well documented in previous studies [25]. While CS group’s performance clearly worsens in EC condition, SS group shows poor balance in both EC and EO conditions. It is well known that visual information is an important component of balance even during quiet stance, as evidenced by the fact that both the amplitude and variability of body sway increase during EC condition [12-16]. The results of this study indicate that in SS subjects visual information did not improve balance performance as much as in healthy subjects. The control of upright posture is a complex mechanism that involves the continuous integration of afferent signals from the visual, vestibular and somatosensory systems [14,15]; and it requires intact effectors in order to realize the correct postural program. Individuals who have experienced injury to the central nervous system in the form of a stroke may exhibit difficulty with sensory processing and/or motor planning. In these patients, the inability of peripheral sensory receptors to gain information about the environment may result in impaired postural control. Results might provide evidence that subjects affected by stroke rely on their vestibular and proprioceptive system in a greater degree than healthy subjects, who rely heavily on their visual feedback [26].

CoP path was significantly larger for the SS group in both AP and ML directions in EO and EC conditions, similarly to what reported by Corriveau et al. [31]. Consequently, the difference is clinically significant and confirms the postural instability in both directions (AP, ML) of the SS compared with the group of age-matched CS. In contrast, only the AP component of CoP was significantly different in the EC condition. Similar results were obtained for the CoP velocity, a parameter that is highly correlated to CoP path. In disagreement with Corriveau et al. [31] results on CoP path in SS suggests that patients affected by hemiplegia do not rely primarily on vision to compensate for motor control deficits in the lower extremity.

When analyzing the relation between posturographic analysis and clinical measures in SS subjects, the TUG scale showed the greater amount of correlation to CoP parameters (sway area, CoPv, CoPv in the AP direction for both the EO and EC condition; CoP path in the AP direction and EO condition; CoPv in the ML direction and EC condition). BBT was also correlated to CoP parameters, although to a lesser degree (CoP path in the AP direction and EO condition; CoPv and CoPv in the ML direction and EC condition; CoPv in the AP direction in both the EC and EO conditions). TB was never correlated to posturographic parameters. These results are in agreement with those of Corriveau et al. [31], who showed significant correlation between CoP-Center of mass amplitude and balance scales (BBS, Tinetti scale). Only one study [30] compared clinical evaluation with laboratory measures in a stroke population maintaining a quiet standing position in EO. BBS was compared with CoP speed, CoP root-mean-square (RMS) value, and CoP mean frequency in the AP and ML directions. In the AP direction, their results were comparable (R^2^ range, 0.50 to 0.57) to ours (R^2^ = 0.56). Surprisingly, significant correlations were not found in the ML direction.

An interesting result of our study is the correlation found between CoP path in the ML direction (R^2^ = 0.69) with the evaluation of the functional walking time measured by the TUG test. Also note that in a recent study, the TUG test proved to be a valid measure for predicting falls [12] as well as functional daily activity in elderly SS [12].

The correlation between functional evaluations and instrumental measures suggests that some of the CoP parameters provide an indication of postural instability in a quasi-static position that is also provided by functional tests used in a dynamic clinical evaluation. However, not all the CoP variables and the functional outcomes were correlated, and often only moderately. This results might indicate that the two techniques provide information about different aspects of balance. However, the precise balance characteristics described by functional balance evaluations are not easy to define, since a measure of deficit can never be perfectly related to a measure of incapacity because other factors enter into play to reduce performance. Certainly, the outcomes of instrumented posturography are useful to understand how a sensorimotor deficit results in functional limitations due to balance problems. In this respect, posturography is an essential tool in understanding the risk of falls [8,23,31].

Rehabilitations services are largely provided during the post-acute phase of a stroke and therapists and physiatrists document the clinical manifestations of stroke in order to select appropriate rehabilitation treatment [32]. The treatments are generally focused on optimizing SS motor performance by means of postural control exercises in order to diminish maladaptive strategies and promote increase loading of the affected lower limb, encourage reactive and anticipatory postural control strategies when displacement of center of mass increases [33]. With this in mind, the present results may indicate a specific role of CoP measurement in identifying those patients who will most likely benefit from rehabilitation and in identifying the more appropriate rehabilitation protocols.

Our results suggest that combining quantitative posturography and clinical evaluation whenever possible would enhance comprehension of postural impairments and disabilities in SS patients.

## Conclusions

This study provides the first attempt at finding a correlation between clinical and instrumental measures of balance in post-stroke subjects, understanding their individual and combined usefulness.

The observation that only some clinical and instrumental balance assessments are related might indicate that they measure different aspects of balance. Consistently with previous findings in healthy and pathologic subjects [12-16], results suggest that posturography parameters were found to provide insight into the postural control mechanisms of post-stroke subjects. Thus, this methodology should be recommended for use in clinical practice. As it has been previously demonstrated in other pathologies (e.g. Parkinson disease, see [16]) post-stroke subjects could also take advantage from the inclusion of quantitative posturography in their balance assessment. Our results may lead to a step forward towards the recommendation of the CoP parameters for use in clinical practice and in research.

Additional investigations are necessary to understand specificity and reliability of the individual center of pressure measures and to further clarify whether they are good candidate measures to discriminate among postural strategies used by post-stroke subjects.

## Competing interests

Each of the authors has read and concurs with the content in the final manuscript. The contributing authors guarantee that this manuscript has not been submitted, nor published elsewhere. Each of the authors declares that don’t have any financial and non-financial competing interests.

## Authors’ contributions

Each of the authors has read and concurs with the content in the final manuscript. ZS, EC, SM and CC participated in conceiving the study. ZS, EC, PC, SM and CC participated in its design and coordination and carried out the drafting of the manuscript. PC helped to draft the manuscript. ZS, PC, AG carried out the experimental part of the study relatives to centre of pressure data collection and carried out and coordinated the data analysis. ZS performed the data analysis. EC carried out the experimental part of the study relatives to the clinical evaluation and participated to the center of pressure data collection. EC made the diagnosis of SS, followed the treatment and supervised the manuscript. All authors read and approved the final manuscript.

## References

[B1] The Italian guidelines for stroke prevention and treatment (SPREAD) 2007 Milano: Ed. Hypephar Group

[B2] Truelsen T Piechowski-Jozwiak B Bonita R Mathers C Boquosslavsky J Boysen G Stroke incidence and prevalence in Europe: a review of available data Eur J Neurol 2006 13 581 598 10.1111/j.1468-1331.2006.01138.x 16796582

[B3] Gryfe CI Amies A Ashley MJ A longitudinal study of falls in an elderly population: incidence and morbidity Age Ageing 1977 6 201 210 10.1093/ageing/6.4.201 596307

[B4] Wild D Nayak USL Isaacs B Prognosis of falls in old people living at home J Epidemiol Comm Health 1981 35 200 204 10.1136/jech.35.3.200 PMC1052157 7328380

[B5] Baker SP Harvey AH Fall injuries in the elderly Clin Geriatr Med 1985 1 501 512 3913506

[B6] Tinetti ME Performance-oriented assessment of mobility in elderly patients J Am Geriatr Soc 1986 34 119 126 3944402 10.1111/j.1532-5415.1986.tb05480.x

[B7] Tinetti ME Speechely M Ginter SF Risk factors for falls among elderly persons living in the community N Engl J Med 1988 319 1701 1707 10.1056/NEJM198812293192604 3205267

[B8] Nyberg L Gustafson Y Patient falls in stroke rehabilitation. a challenge to rehabilitation strategies Stroke 1995 26 838 427 10.1161/01.STR.26.5.838 7740577

[B9] Yavuzer G Eser F Karakus D Karaoglan B Stam HJ The effects of balance training on gait late after stroke: a randomized controlled trial Clin Rehab 2006 20 960 969 10.1177/0269215506070315 17065539

[B10] Benvenuti F Mecacci R Gineprari I Bandinelli S Benvenuti E Ferrucci L Baroni A Rabuffetti M Hallett M Dambrosia JM Stanhope SJ Kinematic characteristics of standing disequilibrium: reliability and validity of a posturographic protocol Arch Phys Med Rehabil 1999 80 278 287 10.1016/S0003-9993(99)90138-7 10084435

[B11] Franchignoni F Tesio L Benevolo E Ottonello M Psychometric properties of the Rivermead Mobility Index in Italian stroke rehabilitation inpatients Clin Rehabil 2003 17 273 282 10.1191/0269215503cr608oa 12735534

[B12] De Oliveira CB de Medeiros IR T Ferreira Frota NA Greters ME Conforto AB Balance control in hemiparetic stroke patients: main tools for evaluation J Rehab Res Devel 2008 45 1215 1226 10.1682/JRRD.2007.09.0150 19235121

[B13] Chiari L Cappello A Lenzi D Della Croce U An improved technique for the extraction of stochastic parameters from stabilograms Gait Posture 2000 12 225 234 10.1016/S0966-6362(00)00086-2 11154933

[B14] Prieto TE Myklebust JB Hoffmann RG Lovett EG Myklebust BM Measures of postural steadiness: differences between healthy young and elderly adults IEEE Trans Biomed Eng 1996 43 956 966 10.1109/10.532130 9214811

[B15] Horak FB Henry SM Shumway-Cook A Postural perturbations: new insights for treatment of balance disorders Phys Ther 1997 77 517 533 9149762 10.1093/ptj/77.5.517

[B16] Rocchi L Chiari L Cappello A Horak FB Identification of distinct characteristics of postural sway in Parkinson's disease: a feature selection procedure based on principal component analysis Neurosci Lett 2006 394 2 140 145 10.1016/j.neulet.2005.10.020 16269212

[B17] Podsiadlo D Richardson S The timed “Up & Go”: a test of basic functional mobility for frail elderly persons J Am Geriatr Soc 1991 39 142 148 1991946 10.1111/j.1532-5415.1991.tb01616.x

[B18] Schmid M Conforto S Camomilla V Cappozzo A D’Alessio T The sensitivity of posturographic parameters to acquisition settings Med Eng Phys 2002 24 623 631 10.1016/S1350-4533(02)00046-2 12376049

[B19] Fugl-Meyer AR Jääskö L Leyman I Olsson S Steglind S The post-stroke hemiplegic patient. 1. a method for evaluation of physical performance Scand J Rehab Med 1975 7 13 31 1135616

[B20] Demeurisse G Demol O Robaye E Motor evaluation in vascular hemiplegia Eur Neurol 1980 19 382 389 10.1159/000115178 7439211

[B21] Collin C Wade D Assessing motor impairment after stroke: a pilot reliability study J Neurol Neurosurg Psychiatry 1990 53 576 579 10.1136/jnnp.53.7.576 2391521 PMC488133

[B22] Tesio L Granger CV Perucca L Franchignoni FP Battaglia MA Russell CF The FIM instrument in the United States and Italy: a comparative study Am J Phys Med Rehabil 2002 81 168 176 10.1097/00002060-200203000-00003 11989512

[B23] Functional Independence Measure: versione Italiana. Manuale d’uso Ricerca in Riabilitazione 1992 2 Suppl 1 44

[B24] Kidd D Stewart G Baldry J Johnson J Rossiter D Petruckevitch A Thompson AJ The Functional Independence Measure: a comparative validity and reliability study Disabil Rehabil 1995 17 10 14 10.3109/09638289509166622 7858276

[B25] Bogle Thorbahn LD Newton RA Use of the Berg balance test to predict falls in elderly persons Phys Ther 1996 76 576 583 8650273 10.1093/ptj/76.6.576

[B26] Horak FB Clinical measurement of postural control in adults Phys Ther 1987 67 1881 1885 3685116 10.1093/ptj/67.12.1881

[B27] Masiero S Avesani R Armani M Postal V Ermani M Predictive factors for ambulation in stroke patients in the rehabilitation setting: a multivariate analysis Clin Neurol Neurosurg 2007 109 9 763 769 10.1016/j.clineuro.2007.07.009 17766038

[B28] Bourdiol JR Pied et statique 1980 Moulin- les-Metz: Maisonneuve Press

[B29] Sawacha Z Carraro E Del Din S Guiotto A Bonaldo L Punzi L Cobelli C Masiero S Biomechanical assessment of balance and posture in subjects with ankylosing spondylitis JNER 2012 9 1 63 10.1186/1743-0003-9-63 22931459 PMC3517897

[B30] Berg K Wood-Dauphinee S Williams JI Maki B Measuring balance in the elderly: validation of an instrument Can J Pub Health 1992 2 S7 S11 1468055

[B31] Corriveau H He’bert R Raîche M Prince F Evaluation of postural stability in the elderly with stroke Arch Phys Med Rehabil 2004 85 1095 1101 10.1016/j.apmr.2003.09.023 15241756

[B32] Zajdel K Latała B Mosurska D The usefulness of posturography and caloric tests in selected neurological diseases Przegl Lek 2009 66 920 993 20297629

[B33] Carr JS R Neurological rehabilitation: Optimizing motor performance 1998 Woodburn: Butterworth Heinman

